# The T1048I mutation in *ATP7A* gene causes an unusual Menkes disease presentation

**DOI:** 10.1186/1471-2431-12-150

**Published:** 2012-09-19

**Authors:** Gregorio León-García, Alfredo Santana, Nicolás Villegas-Sepúlveda, Concepción Pérez-González, José M Henrríquez-Esquíroz, Carlota de León-García, Carlos Wong, Isabel Baeza

**Affiliations:** 1 Department of Biochemistry, National School of Biological Sciences, National Polytechnic Institute (IPN), Mexico City, 11340, Mexico; 2 Centre for Biomedical Research on Rare Disease (CIBERER), Canarias University Hospital, Institute of Biomedical Technologies, University of La Laguna, Tenerife, Spain; 3 Department of Molecular Biology, Centre for Research and Advanced Studies, IPN, Mexico City, 07360, Mexico; 4 Department of Paediatrics, Jose Molina-Orosa Hospital, Lanzarote, 35500, Spain; 5 Early Care and Child Development Centre, Lanzarote, 35500, Spain

**Keywords:** ATP7A, Menkes disease, Copper transporter, Cu-His treatment

## Abstract

**Background:**

The *ATP7A* gene encodes the ATP7A protein, which is a trans-Golgi network copper transporter expressed in the brain and other organs. Mutations in this gene cause disorders of copper metabolism, such as Menkes disease. Here we describe the novel and unusual mutation (p.T1048I) in the *ATP7A* gene of a child with Menkes disease. The mutation affects a conserved DKTGT^1048^ phosphorylation motif that is involved in the catalytic activity of ATP7A. We also describe the clinical course and the response to copper treatment in this patient.

**Case presentation:**

An 11-month-old male Caucasian infant was studied because of hypotonia, ataxia and global developmental delay. The patient presented low levels of serum copper and ceruloplasmin, and was shown to be hemizygous for the p.T1048I mutation in ATP7A. The diagnosis was confirmed when the patient was 18 months old, and treatment with copper-histidinate (Cu-His) was started immediately. The patient showed some neurological improvement and he is currently 8 years old. Because the p.T1048I mutation affects its catalytic site, we expected a complete loss of functional ATP7A and a classical Menkes disease presentation. However, the clinical course of the patient was mild, and he responded to Cu-His treatment, which suggests that this mutation leads to partial conservation of the activity of ATP7A.

**Conclusion:**

This case emphasizes the important correlation between genotype and phenotype in patients with Menkes disease. The prognosis in Menkes disease is associated with early detection, early initiation of treatment and with the preservation of some ATP7A activity, which is necessary for Cu-His treatment response. The description of this new mutation and the response of the patient to Cu-His treatment will contribute to the growing body of knowledge about treatment response in Menkes disease.

## Background

Menkes disease (MD) (OMIM #309400) is an X-linked recessive disorder of copper metabolism; it is caused by mutations in the *ATP7A* gene [1]. There are several clinical variants of MD: classical MD (90–95% of patients), mild MD, occipital horn syndrome [2] and a new syndrome characterized by isolated distal motor neuropathy, no signs of copper deficiency and adult onset [3]. Classic MD is characterized by neonatal neurological degeneration, coarse and twisted hair, hypopigmentation and seizures; death usually occurs by the age of three years [2]. Mild MD is a moderate form of the disease, in which cerebellar ataxia and moderate developmental delay predominate [4]. Occipital horn syndrome is the mildest form of MD and is characterized by connective tissue abnormalities and some neurological alterations [2]; in this form of the disease, the ATP7A protein retains some of its activity [5,6].

**Figure 1:**
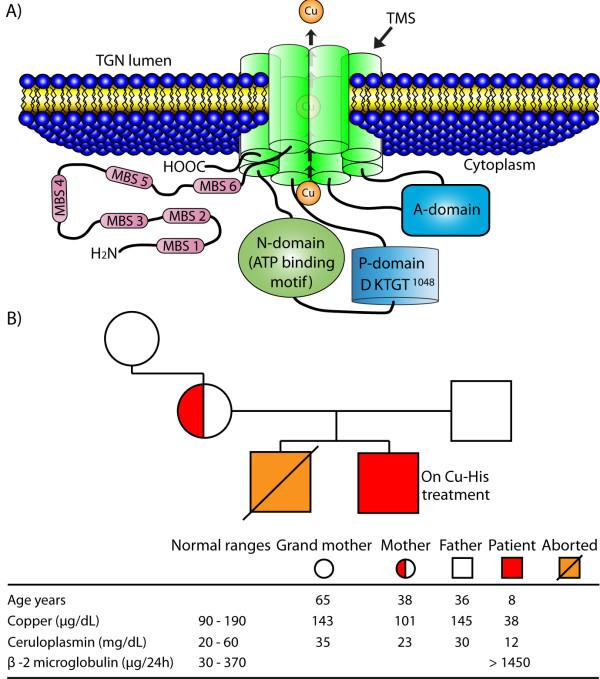
**A) Transmembrane organization of the human ATP7A protein.** This schematic representation is based on structural studies of ATP7A [7]. ATP7A contains five regions: i) an N-terminal tail with six metal-binding sites (MBS 1–6), ii) eight trans-membrane segments (TMS), iii) an ATP-binding domain that contains a nucleotide-binding motif (N-domain) and a phosphorylation motif (P-domain, DKTGT^1048^), iv) an A-domain and v) a C-terminal tail. **B)** Presence of ATP7A gene mutation (c.3288 C > T), and copper, ceruloplasmin and β-2 microglobulin levels in the patient and in members of his family. The patient’s serum levels of copper and ceruloplasmin were measured while he was receiving Cu-His (100 μg/kg/day). The urine level of β-2 microglobulin was determined 6.5 years after the administration of Cu-His (normal range, 30–370 μg/24 h).

The *ATP7A* gene encodes a transmembrane P-type ATPase that delivers copper to copper-dependent enzymes in the trans-Golgi network (TGN). The P-type ATPases ATP7A and ATP7B are membrane proteins that hydrolyse ATP for the active transport of cations across cellular membranes, forming acyl-phosphate intermediates [7]. The loss of ATP7B function causes Wilson’s disease [8], while mutations that eliminate or reduce the activity of ATP7A cause MD. ATP7A resides in the TGN but it can relocate to the basolateral membrane of polarized cells in response to increased extracellular copper concentrations [9]; this protein contains an N-terminal tail with six metal-binding sites, eight transmembrane segments, an ATP-binding domain, an A-domain, and a C-terminal tail. The metal-binding sites accept copper from cytoplasmic carriers and deliver it to the channel formed by the transmembrane segments for its transport across the membrane into the TGN. The ATP-binding domain contains a nucleotide-binding motif (N-domain) and a phosphorylation motif (P-domain); the phosphorylation motif (DKTGT) starts on the 1044 aspartate residue (D^1044^) [7], it is conserved in all P-type ATPases [10] and participates in the catalytic reaction as the acyl-phosphate intermediate. The A-domain is a phosphatase that hydrolyses the acyl-phosphate after copper transport [7,11] (Figure 1A). In this study, we report a mutation that affects the phosphorylation motif (DKTGT^1048^) of human ATP7A in an infant with MD; this mutation involves the substitution of the 1048 threonine residue with an isoleucine residue (p.T1048I). We also describe the clinical course and the response to copper treatment in this patient.

## Case presentation

An 11-month-old male Caucasian infant was referred to the Hospital of Lanzarote as a result of hypotonia (which was observed soon after his birth), ataxia, global developmental delay and clonic seizures. The infant was born after an uncomplicated full-term pregnancy, with a birth weight of 2.5 kg. At the time of examination, he presented psychomotor retardation, hair changes (scarce, thin, coarse), severe head lag and inability to sit independently. The serum concentrations of glucose, urea nitrogen, creatinine, lactate, K^+^, Na^+^, Cl^-^, Ca^2+^ Mg^2+^, phosphorus, liver enzymes, pancreatic amylase, total cholesterol, HDL, LDL, triglycerides and total protein were normal. A diagnosis of MD was suggested by the clinical features of the patient; this diagnosis was supported by reduced levels of copper (38 μg/dl) and ceruloplasmin (12 mg/dl) in serum (normal range, 90–190 μg/dl and 20–60 mg/dl, respectively), and by copper accumulation (determined by atomic absorption spectroscopy) in fibroblasts obtained from the patient and cultured *in vitro* (227 ng/mg protein; normal range, 21–46 ng/mg). The patient’s mother and grandmother had normal copper and ceruloplasmin serum levels (Figure 1B).

**Figure 2:**
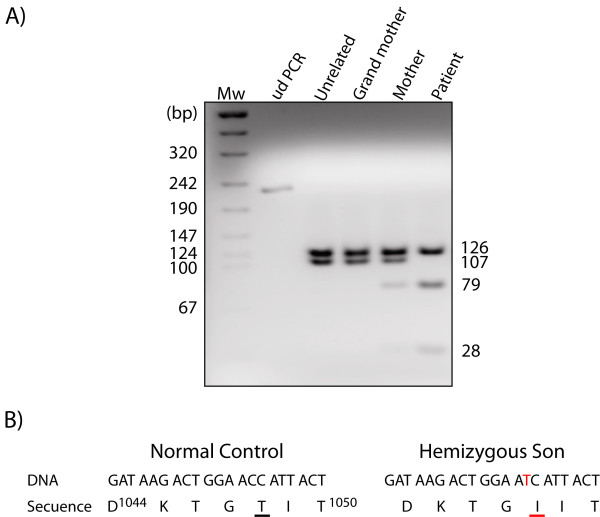
**A) Restriction fragment length polymorphism (RFLP) assay.** Exon 16 of the *ATP7A* gene was amplified using specific primers, and the PCR product was digested with *Hinf*I. The c.3288 C > T mutation introduces a *Hinf*I restriction site that divides the 107 bp fragment into two fragments of 79 and 28 bp. ud PCR, undigested PCR. **B)** DNA sequence of part of the *ATP7A* gene (exon 16) from the patient and from a normal control; the mutation is shown in red.

The patient’s family history was not suggestive of an X-linked disorder; however, his mother had a previous spontaneous abortion. Mutation analysis of the *ATP7A* gene (which is found on chromosome Xq13.2-q13.3) by PCR and DNA sequencing showed that the patient and his mother carried a point mutation in the 16^th^ exon of this gene: a change of C for T (c.3288 C > T), which results in the replacement of the 1048 threonine residue with an isoleucine residue [12,13] (Figure 2B). A specific restriction fragment length polymorphism (RFLP) assay using *Hinf*I corroborated the presence of the p.T1048I mutation in the patient and in his mother, but not in his grandmother (Figure 2A).

Treatment with 100 μg/kg/day of copper-histidinate (Cu-His) (Carreras Pharmaceutical Laboratories, Barcelona, Spain) was initiated when the patient was 18 months old and was maintained for 6.5 years. Cu-His treatment was suspended when an increased β-2 microglobulin concentration was found in the patient’s urine; nephrotoxicity caused by copper treatments is usually reversible [14]. The Cu-His treatment increased copper and ceruloplasmin serum levels to normal values (102 μg/dl and 28 mg/dl, respectively) after three years. Currently, the patient is 8 years old; his hair characteristics and muscular tone have improved, and the frequency of his seizures has decreased. Some partial ATP7A activity is necessary to achieve a response to Cu-His treatment in MD [14]. The mild phenotype presented by this patient and his long survival, together with his response to Cu-His treatment, suggest that the p.T1048I mutation causes only a partial loss of ATP7A function, which probably does not completely block copper transport across the blood–brain barrier and therefore results in moderate neurological impairment.

Only one other mutation in the DKTGT^1048^ motif has been reported: it involves the substitution of the 1044 aspartate residue with a glycine residue (p.D1044G), and it was identified in a patient with classic MD [15]; this mutation completely eliminates ATP7A activity. In ATP7B, the mutation of D1027 (the residue analogue to D1044) prevents the formation of the acyl-phosphate intermediate, and is associated with a complete loss of the copper-transport activity of ATP7B in Wilson’s disease [16,17]. ATP7A mutations in K1045, T1046 or G1047 have not been identified in MD patients; however, directed mutagenesis studies with P-type ATPases report that mutations in analogous residues decreased the affinity of the P-domain for ATP and disrupted the formation of acyl-phosphate [18,19]. Molecular-modelling analysis of ATP bound to the N-domain of ATP7B [20], to the P-domain of Ca^2+^ ATPase and to CopA (a bacterial orthologous Cu^+^-transporting P-type ATPase) [21,22], suggests that the D1044, K1045 and T1046 residues of the P-domain of ATP7A may form hydrogen bonds with the phosphate tail of ATP. The analyses also rule out the participation of the T1048 residue of ATP7A in these hydrogen bonds with ATP, but T1048 could still form hydrogen bonds with amino acid residues in the N- and A-domains of ATP7A.

In the case of p.T1048I (the mutation reported in this study), the replacement of the 1048 threonine residue with isoleucine, an amino acid of different size, charge and hydrophobicity, could alter the interactions between the P-domain and the N- and A-domains, producing an inadequate folding that could affect the formation of the acyl-phosphate intermediate. This altered enzymatic activity may interfere with the copper-induced re-localization of ATP7A from the TGN to the plasma membrane, affecting copper transport and leading to mild MD. This conclusion is supported by mutations of an analogous residue (T1031) in ATP7B, which affect copper transport and are found in patients with Wilson’s disease [23-25]. According to the studies cited above, and to the characteristics of the mutation reported in this study, we propose that mutations in amino acid residues that are involved directly in the attachment and hydrolysis of ATP, in the formation and hydrolysis of acyl-phosphate and in the attachment of copper (such as D1044, K1045, T1046 and G1047) may cause a complete loss of functional ATP7A and result in classical MD. In contrast, mutations in amino acid residues that are not involved in these processes (such as T1048) may cause only a partial loss of function.

Treatment with Cu-His did not completely normalize the neurological manifestations in our patient, but some of them did improve. His epilepsy pattern is multifocal and myoclonic, as was previously reported for MD patients with this age [26,27]. We observed a decrease in the frequency of seizures and ataxic movements, and an increase in motor activities and muscle tone, (which increased from a score of 2 to 3 of a maximum of 5 in the Daniels–Worthingham scale). We also observed significant improvements in the patient’s cognitive and psychosocial functions, but the patient does not speak and he attends a special school for children with different capabilities. The Cu-His treatment was initiated as soon as the diagnosis was confirmed, and by then the patient was 18 months old. In most cases, the neurological improvements of patients with MD that start their treatment so long after birth are poor [28]; however, it is advisable to administer the copper treatment anyway, because it prolongs survival, reduces the frequency of seizures and improves the patient’s quality of life [29,30].

## Conclusion

The p.T1048I mutation, which affects the conserved DKTGT^1048^ phosphorylation motif of ATP7A, probably causes a partial loss of the function of this protein. The residual activity of ATP7A allowed a response to Cu-His treatment in this patient; and although the treatment was started when the patient was 18 months old, it has led to the patient's long survival. This case emphasizes the important correlation between genotype and response to Cu-His treatment in patients with MD. The prognosis in these patients is associated with an early detection of the disease (ideally through detailed newborn screening) and an immediate initiation of Cu-His treatment in eligible patients, before the occurrence of irreversible neurodegeneration.

## Abbreviations

Cu-His: Copper-histidinate; MD: Menkes disease; PCR: Polymerase chain reaction; RFLP: Restriction fragment length polymorphism; TGN: Trans-Golgi network.

## Competing interests

The authors declare that they have no competing interests.

## Authors’ contributions

All authors contributed to conception and design, data acquisition, analysis or interpretation, and gave approval of the final version to be published. GLG and CW designed the study, oversaw the biochemical analysis, and wrote the first draft of the manuscript. AS and NVS carried out the molecular studies, assisted in the interpretation of the results and contributed significantly to the final draft. CPG, JMHE, and CLG provided long-term specialized care to the patient, evaluated the patient, participated in the acquisition and analysis of data, and helped to draft the manuscript. IB reviewed the manuscript for important intellectual content and gave final approval of the version to be published.

## Pre-publication history

The pre-publication history for this paper can be accessed here:

http://www.biomedcentral.com/1471-2431/12/150/prepub
